# Notes and News

**Published:** 1901-01-26

**Authors:** 


### NOTES AND NEWS.

HOSPITAL MEETINGS.

Italian Hospital, Queen Sq.—Annual general meeting, Saturday, January 26th, 4.30.

IT is good news for the Prince of Wales's Fund that the late Mr. S. Lewis has decreed that ultimately it is to become the possessor of no less a sum than £250,000. Further, Mr. S. Lewis showed his appreciation of the institution by an immediate bequest of £500. He has left the same sum to Guy's Hospital, and the Jews' Hospital and Orphan Asylum, besides several Jewish charities. Numerous hospitals and charities are also constituted residuary legatees.

MR. S. LEWIS's magnificent recognition of the work which the Prince of Wales's Hospital Fund for London is doing for the hospitals must be very gratifying to the Prince of Wales and those who are co-operating with him in the scheme. At this time we can none of us forget that the establishment of this Fund was chosen by the Queen herself to commemorate her Diamond Jubilee. Her wish has been well responded to and her judgment justified. Although there are many admirable funds collecting for our hospitals none unites in itself the particular attributes of the Prince of Wales's Hospital Fund, which is non-sectarian, and which appeals to the whole of the population, rich and poor. The Prince himself sets an example of deep and earnest care in the allocation of the funds, coupled with the greatest impartiality and true desire to further all the best principles of administration for the well-being of the sick poor. A keen business man like Mr. S. Lewis was well able to appreciate the desirability of placing the responsibility of dispensing a generous and munificent bequest in hands well able through knowledge and experience to do so.

WE regret to record the death of Dr. Archibald Jacob of Dublin. Dr. Jacob was educated at and an M.D. of the University of Dublin. He served on the medical staff of vari us hospitals in the City, was Professor of Ophthalmology to the Irish Royal College of Surgeons, oculist to the Lord Lieutenant of Ireland, and chief editor and proprietor of the "Medical Press and Circular."

AN epidemic of smallpox, which has not as yet assumed large proportions, is in progress in Glasgow.

AT the meeting of the Metropolitan Hospital Sunday Fund on January 20th, the Distribution Committee for the year was elected. All the gentlemen serving on last year's committee were re-elected.

A SANATORIUM entitled Nordrach-on-Dee for consumptives has been opened at Banchory in Scotland. It is built in the middle of a pine wood on a southern slope. The cost per bed for the establishment has been £600. It is designed for 36 patients, 25 being already in residence.

IN connection with the Church Missionary Society a medical training home for Women Missionaries has been opened at Bermondsey by Lady Chichester. Besides its educational intention the Home is to carry on medical work also amongst the sick poor. There is a resident lady doctor, a superintendent, and a nurse. The nurse is prepared to visit the patients in their own homes, and a dispensary is to be open five times a week.

IT is with genuine regret that we have to report the death of Dr. Sidgwick Saunders, who for so many years has rendered such important public service as medical officer of Health and Public Analyst for the City of London. To him is due the credit of maintaining so high a standard of excellence in regard to the food supplies distributed to Londoners through the Central Markets. In his earlier years he was attached to the Army medical staff, and his writings upon the Sanitary Condition of London and its Water, and especially his reports upon the insanitary state of St. Bartholomew's and Christ's Hospitals, have been fruitful in results. For a quarter of a century Dr. Sidgwick Saunders was an active member of the Distribution Committee of the Hospital Sunday Fund, and he will be much missed on the Council of that body. A staunch friend, a capable and popular practitioner, and a pleasant personality, Dr. Sidgwick Saunders will be long regretted by a host of friends in many stations of life.

ANOTHER Victoria Cross has come to the Royal Army Medical Corps. On February 24th Lieutenant Edgar T. Inkson, R.A.M.C., carried a wounded officer for three or four hundred yards, under a very heavy fire, to a place of safety, the ground over which he had to move being much exposed, and there being no cover available. For this and other acts of gallantry at Colenso and Spion Kop he has been awarded the Victoria Cross. We congratulate him most heartily upon having won so early in his career this coveted distinction, and we congratulate his corps upon this further proof that upon the field of battle it is second to none. Lieutenant Edgar Inkson is the son of a late P.M.O. at Portsmouth. He studied medicine at University College, London, and during the time that he was there he was a member (we believe a staff sergeant) of the Volunteer Medical Staff Corps. Major Baptie of the same corps, who has also been a recipient of the Victoria Cross, has been presented with the freedom of the burgh of Dumbarton. Mr. Donald McDowell, principal medical officer of the West African Frontier Force, who lately served with the Ashanti Field Force, has been created a Companion of the Order of St. Michael and St. George in recognition of his services.

